# Whether Screening for Non-alcoholic Fatty Liver Disease in Patients With Psoriasis Is Necessary: A Pilot Quality Improvement Project

**DOI:** 10.7759/cureus.24714

**Published:** 2022-05-03

**Authors:** Kader Torbator, Stephanie Poo, Taif Al-Rubaye, Leah Mapara, Sungeeta Punjabi, Ali Al-Rubaye, Laith Alrubaiy

**Affiliations:** 1 Gastroenterology and Hepatology, London Northwest NHS Trust, London, GBR; 2 General Practice, Manchester NHS Trust, Manchester, GBR; 3 Dermatology, London Northwest NHS Trust, London, GBR; 4 Public Health, Basra Health Directorate, Basra, IRQ; 5 Gastroenterology, St Mark's Hospital, London, GBR

**Keywords:** transient elastography (fibroscan), quality improvement, cirrhosis, non-alcoholic fatty liver disease, psoriasis

## Abstract

Background

Psoriasis is a chronic inflammatory skin disease that is strongly associated with non-alcoholic fatty liver disease (NAFLD). Both conditions are associated with excess cardiovascular and liver-related morbidity and mortality. The severity of psoriasis correlates with the degree of liver inflammation and scarring, which can be further exacerbated by systemic immunomodulators such as methotrexate.

Currently, no clinical pathway exists to screen psoriasis patients for NAFLD in our Trust. We aimed to develop a shared clinical pathway between our hepatology and dermatology departments to allow early identification and management of NAFLD in this patient group.

Methods

A multidisciplinary team was assembled to identify patient priorities, management goals, and screening criteria. We identified gaps in our service and reviewed current clinical best practice guidelines. A clinical pathway was developed using a process map and revised according to feedback received. We piloted this pathway on a prospective cohort of psoriasis patients identified by our dermatology department. Patients were invited for transient elastography if fatty liver was identified on an ultrasound scan. Baseline demographics, biochemistry and imaging results were collected and analysed.

Results

Of 57 psoriasis patients, 30 (52.6%) had sonographic evidence of hepatic steatosis. The median age was comparable between groups with 56 and 55 years in the psoriasis-NAFLD (Ps-NAFLD) and no-NAFLD groups respectively. There were more males in the Ps-NAFLDgroup (56.7%) compared to the no-NAFLD group (37%). Fifteen out of 30 patients were eligible for transient elastography (two were excluded due to body habitus). Seven (53.8%) patients had no-to-mild fibrosis indicated by liver stiffness measurement (LSM) *≤*7kPa, while six (46.1%) had moderate-to-severe fibrosis. Three (23.0%) patients had scores suggestive of cirrhosis (LSM>13kPa).

Conclusions

The introduction of a new shared-care pathway at our Trust has resulted in a streamlined way in which psoriasis patients can be screened and treated for NAFLD.

## Introduction

Non-alcoholic fatty liver disease (NAFLD) is an increasing public health concern resulting in a growing burden of morbidity- and liver-related complications and mortality worldwide [1]. Psoriasis is a chronic immune-mediated inflammatory skin condition that is strongly associated with non-alcoholic fatty liver disease, with 70% of patients more likely to develop NAFLD and advanced liver fibrosis, independent of other risk factors [2]. Patients with moderate-severe psoriasis appear to be particularly affected, compounded by the administration of systemic immunomodulators such as methotrexate which are potentially hepatotoxic. Early identification and management are therefore crucial in order to prevent irreversible fibrosis, end-stage liver disease and downstream extrahepatic complications.

Despite the significant associations between psoriasis and NAFLD, no clinical pathway currently exists to screen patients with psoriasis for non-alcoholic fatty liver disease at our NHS Trust. We aim to develop a shared care pathway for early identification and management of NAFLD in psoriasis patients in collaboration with the dermatology and hepatology departments and identify the local prevalence of NAFLD and advanced liver fibrosis in this group.

Non-alcoholic fatty liver disease is a metabolic liver disorder and is a huge public health concern due to the increasing prevalence of the disease and its associated morbidity and mortality [3]. The global prevalence of the disease is rising exponentially and is estimated to increase to 33.5% by 2030 [4]. NAFLD ranges from hepatic steatosis, steatohepatitis and advanced liver fibrosis, and is now thought to be the leading cause of chronic liver disease worldwide.

Psoriasis is associated with NAFLD, and the mechanisms that underpin this bidirectional relationship are complex and multifactorial [5,6]. Both conditions are associated with metabolic syndrome, and it is thought that the chronic pro-inflammatory, pro-atherogenic and pro-oxidant state ultimately result in a vicious cycle of free fatty acid accumulation, chronic hepatic inflammation and fibrogenesis [6-8]. This probably explains why psoriasis patients have more severe liver impairment, and vice versa [2]. In addition, psoriasis patients are often treated with immunosuppressive therapy such as methotrexate which can further exacerbate hepatotoxicity.

Liver biopsy is considered the gold standard method to diagnose liver fibrosis, however, it is invasive and carries a considerable risk of complications [9,10]. Several non-invasive tests, including serum biomarkers and elastography-based techniques, are increasingly adopted in clinical practice [11]. Indeed scoring systems such as the NAFLD-fibrosis and FIB-4 scores have been developed based on serum biomarkers to estimate the risk of development of liver scarring [12,13]. These non-invasive methods are attractive alternatives as elastography measurements can aid prognostication, and repeated assessments can be performed to monitor the progression of the disease.

Our Trust utilises transient elastography (TE) for patients with sonographic evidence of hepatic steatosis and NAFLD fibrosis score of >-1.455 or deranged liver function tests for more than three months, provided other diagnoses have been excluded with a liver screen. Most referrals for NAFLD come from primary care via the North West London Non-Alcoholic Fatty Liver Disease pathway, which adopts similar principles [14].

Despite the establishment of this bundle, there is no clear pathway that allows screening for NAFLD in psoriasis patients, which risks late or missed diagnoses of NAFLD and consequently downstream morbidity and mortality. There is therefore a need for improvement in the timely recognition and management of NAFLD in this patient population.

Given the absence of a streamlined pathway for which psoriasis patients can be screened for NAFLD, the prevalence of NAFLD in our local psoriasis population is not known. We planned to initiate a screening pathway in a pilot patient group and collect baseline demographic data.

## Materials and methods

**Figure 1:**
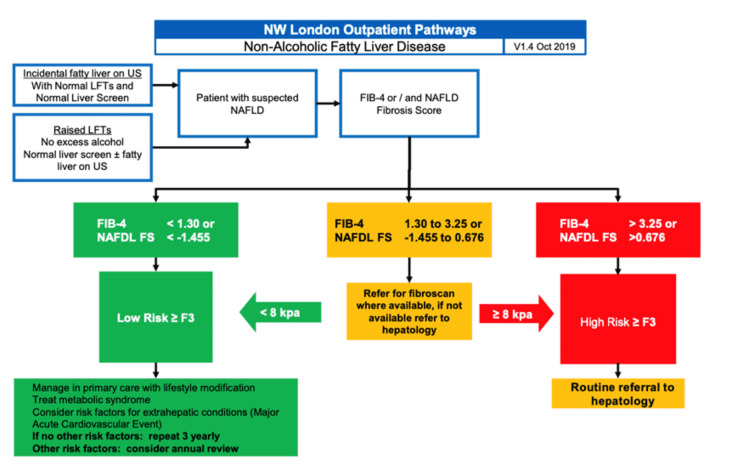
North West London clinical commissioning group non-alcoholic fatty liver disease referral pathway for primary care physicians NAFLD: Non-alcoholic fatty liver disease; US: ulstrasound scan; LFT: liver function test; FS: Fibrosis score;

**Figure 2:**
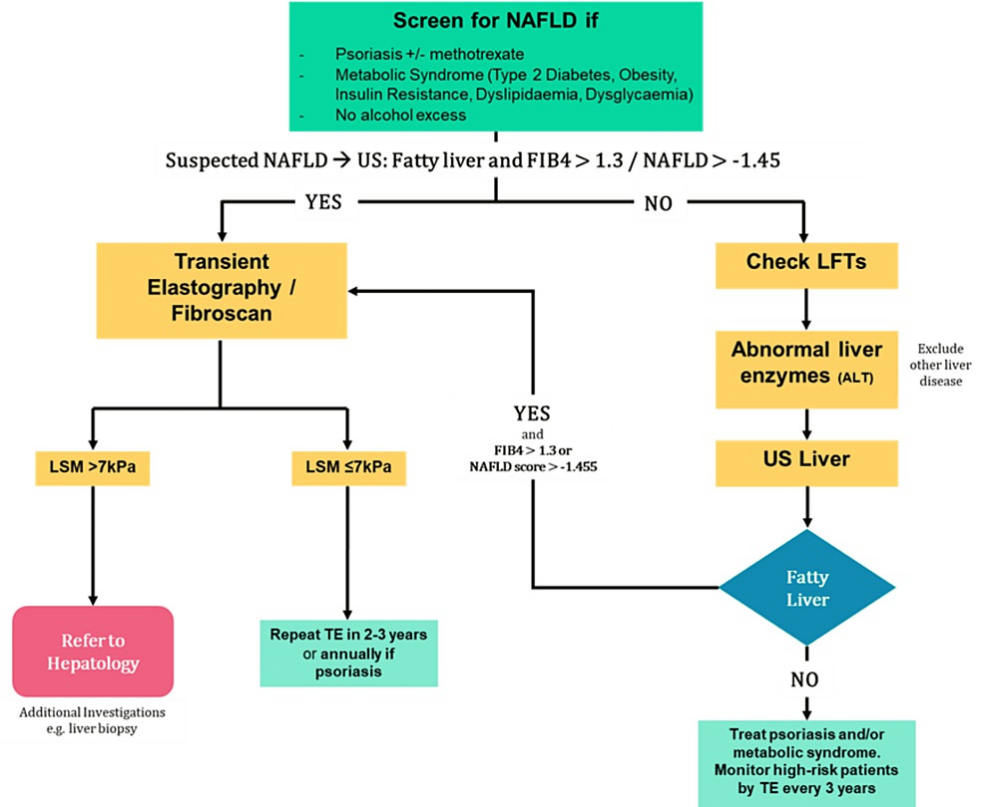
Clinical pathway for screening of patients with psoriasis and other risk factors for NAFLD Abnormal liver enzymes mean ALT greater than two times the upper limit of normal (ULN) LSM, Liver Stiffness Measurement; TE, Transient Elastography; LFTs; Liver Function Tests; US, Ultrasound Scan, NAFLD, Non-Alcoholic Fatty Liver Disease; ALT: Alanine Aminotransferase Adapted from St Johns Institute of Dermatology at Guy’s and St Thomas Hospital NHS Trust protocol.

We assembled a multidisciplinary team comprising dermatologists, hepatologists, radiologists, biochemists and the pharmacy department. The team identified gaps in the service and reviewed the literature, current local clinical practice guidelines as well as the British Association of Dermatology guidelines [14]. In addition, we collaborated with the St Johns Institute of Dermatology at Guys and St Thomas Hospital NHS Trust for their expertise in order to establish a local clinical pathway for screening (Figure 1). We used a process map to demonstrate a clear visual representation of the patient flow and identify any potentially unnecessary steps (Figure 2). Our pathway draws on principles by Wilson and Jungner for the development of a successful screening programme [15]. We discussed the feasibility and sustainability of implementing a streamlined referral pathway between our dermatology, radiology and hepatology departments, taking into account factors such as cost, acceptability and human resources.

We piloted this protocol to trial its implementation on a cohort of psoriasis patients. Adult patients with psoriasis were prospectively identified from our dermatology department. Patients with alcoholic liver disease or incomplete clinical information were excluded. Baseline demographics, biochemistry and imaging results were collected and analysed. Psoriasis patients with radiological evidence of NAFLD on ultrasound scan of the liver, with fatty liver indicated by increased attenuation, reflectivity and echogenicity were invited to the hepatology clinic. Patients with sonographic evidence of hepatic stenosis were offered transient elastography to assess for fibrosis using liver stiffness measurement (LSM). LSM 7kPa indicated no-to-mild fibrosis (F0-1 METAVIR) while LSM >7kPA suggested significant liver fibrosis (F2 METAVIR) [16,17].

## Results

**Figure 3:**
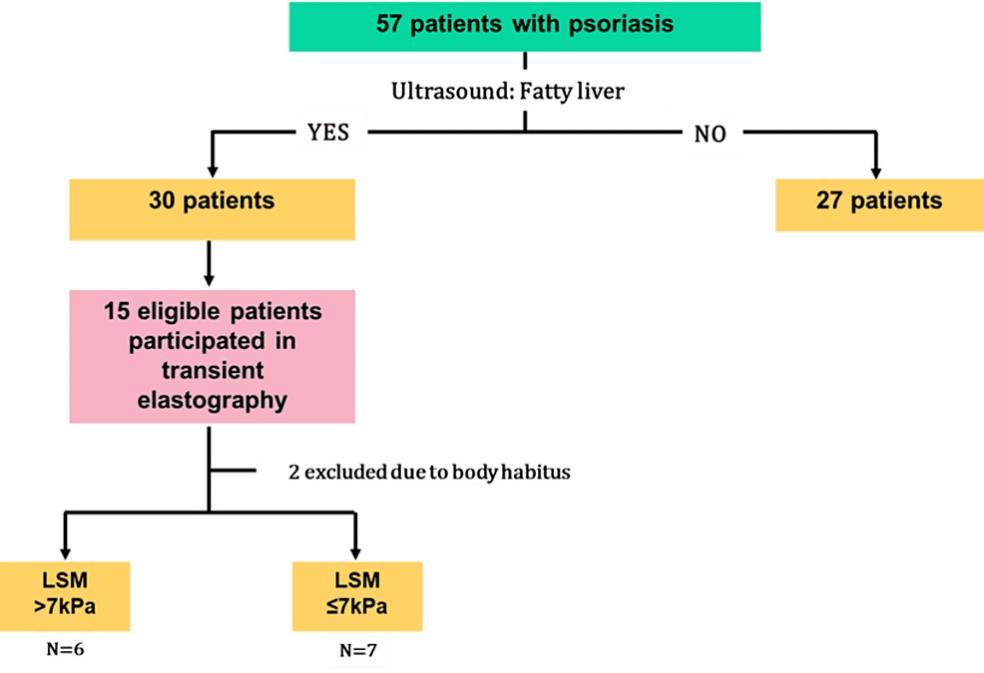
Flow chart of study cohort LSM, Liver Stiffness Measurement

Figure 3 summarises the flow chart of the study cohort. Fifty-seven patients with psoriasis were identified, of whom 30 (52.6%) had fatty liver confirmed on ultrasound. The median age was comparable between groups with 56 and 55 years in the psoriasis-NAFLD (Ps-NAFLD) and no-NAFLD groups, respectively, while there was a higher proportion of males in the Ps-NAFLD group(56.7%) compared to no-NAFLD group (37%) (Table 1). There were no significant differences in the biochemistry between the two groups, except for alanine transaminase levels, which were significantly greater in those with Ps-NAFLD than psoriasis alone (p=0.0008).

**Table 1:** Comparison of patients with psoriasis who had NAFLD versus patients with psoriasis who did not have NAFLD NAFLD: non-alcoholic fatty liver disease

	Patients with Psoriasis and NAFLD (Ps-NAFLD)	Patients with Psoriasis without NAFLD
Numbers	30 ( 52.6%)	27 (47.4%)
Males	17 (56.7 %)	10 (37%)
Females	13 (43.3%)	17 (63%)

Fifteen out of the 30 patients with Ps-NAFLD were able to have transient elastography. However, two patients were excluded due to difficulties in obtaining measurements attributed to their large body habitus. Seven (53.8%) patients had no-to-mild fibrosis (F0-1) indicated by LSM 7kPa. Two (15.4%) had significant fibrosis (≥F2, LSM >7kPa), one (7.7%) had advanced fibrosis (≥F3, LSM >9.5kPa) and three (23.1%) had cirrhosis (F4, LSM>13kPa).

## Discussion

We report the successful implementation of the psoriasis-NAFLD pathway at our Trust in a cohort of psoriasis patients. Over half had fatty liver identified on an ultrasound scan, and of those eligible for transient elastography (TE), six (46.4%) had significant-to-advanced fibrosis. Our data confirm the significant association between psoriasis and NAFLD and justify the importance of screening psoriasis patients for this condition.

The key success in this process was the collaborative and multi-disciplinary approach with key stakeholders. St John’s Institute of Dermatology helped to provide expertise in sharing knowledge and protocol design, while engagement with the dermatology, hepatology and radiology departments, respectively allowed implementation and continuous improvement of our shared care pathway. Process improvements required regular feedback meetings, and the use of process mapping-aided visual clarity and fine-tuning.

With the development of a new screening programme, cost, time and human resources were key factors for consideration. Our Trust currently uses acoustic radiation force impulse (ARFI) elastography which is performed by two expert radiologists to assess the degree of liver scarring. There were concerns that our pathway would lead to a significantly increased workload on our radiology department. In order to address this, a FibroScan has been acquired and a hepatology-led FibroScan service is being established. To facilitate referrals into this service, an electronic requesting system is being developed and will include screening blood tests to calculate scores such as FIB4 or NAFLD scores.

Data from our pilot patient population provided timely feedback on the feasibility of our shared care pathway as well as the prevalence of Ps-NAFLD in our local population.

The main limitation of our study is the small sample size of our pilot patient population which could result in an overestimation of the prevalence of NAFLD among psoriasis patients. Given that no screening pathway exists for psoriasis patients, we did not have any baseline measurements to compare our results with, however, our pilot data supports previously published associations between psoriasis and NAFLD. Data from a larger prospective cohort will provide an accurate representation of the prevalence of NAFLD and liver fibrosis in this group. There is an increasing trend to re-name NAFLD to Metabolic-Associated Fatty Liver Disease (MAFLD). MAFLD is defined as evidence of hepatic steatosis in addition to metabolic dysfunction criteria, this definition would be more suitable in patients with psoriasis and fatty liver disease. For this definition exclusion of steatogenic medication is not a criterion [18]

While transient elastography is a useful non-invasive method for estimating the degree of liver scarring, false positives may occur due to the presence of liver inflammation or congestion in cases of congestive cardiac failure. In addition, failure to obtain accurate measurements may occur as a result of ascites or large body habitus. Given that NAFLD is commonly associated with metabolic syndrome and centripetal obesity, this may present a significant limitation in conducting TE.

Continuous engagement from physicians will be an ongoing challenge, and the success of this pathway relies on proactive monitoring of liver function tests and the identification of high-risk individuals for TE. This can be addressed through an established protocol in our hospital intranet, regular educational meetings, audits and electronic reminders for patients under surveillance.

Finally, patient and staff acceptability were not addressed in our quality improvement project. Subsequent PDSAcycles should include qualitative feedback in the form of patient and physician questionnaires in order to further improve our current psoriasis-NAFLD pathway.

## Conclusions

Here we present our initiative to develop and implement a shared care pathway for psoriasis-NAFLD patients in collaboration with our local hepatology, dermatology and radiology departments. This initiative has allowed screening of NAFLD among psoriasis patients, which has led to the early identification and management of liver disease in this group.
